# BAMLET (Bovine α-lactalbumin made lethal to tumor cells) inhibits autophagy flux and induces apoptosis via down-regulation of protein kinase CK1α and attenuation of the AKT/p-ß-catenin (S552) pathway in RAS-mutated human colorectal HCT 116 cells

**DOI:** 10.22038/IJBMS.2023.69343.15114

**Published:** 2023

**Authors:** Hamid Behrouj, Pooneh Mokarram

**Affiliations:** 1 Department of Biochemistry, School of Medicine, Shiraz University of Medical Sciences, Shiraz, Iran; 2 Behbahan Faculty of Medical Sciences, Behbahan, Iran; 3 Autophagy Research Center, Shiraz University of Medical Sciences, Shiraz, Iran

**Keywords:** AKT/p-ß-catenin, (S552)Autophagy, BAMLET, CK1α, RAS

## Abstract

**Objective(s):**

Oncogenic RAS mutations occur in nearly 50% of colorectal cancer cases and are usually dependent on the autophagy mechanism to maintain tumorigenesis. We have recently demonstrated that CK1α controls autophagy machinery possibly through the AKT/p-ß-catenin (S552) signaling in colorectal cancer cells harboring RAS mutation. It has been found that a lipid-protein complex comprising oleic acid binds to human α-lactalbumin, known as HAMLET (human α -lactalbumin made lethal to tumor cells), targets a broad range of kinases including CK1α. Therefore, this study was designed to investigate the effects of BAMLET (bovine α -lactalbumin made lethal to tumor cells, the bovine counterpart of HAMLET) on CK1α expression, AKT/Phospho-ß-catenin (S552) pathway, and autophagy flux in RAS-mutated human colorectal HCT 116 cells.

**Materials and Methods:**

For this purpose, HCT116 cells were treated with BAMLET and casein kinase 1 inhibitor (D4476), and quantitative real-time polymerase chain reaction (RT-qPCR) and western blot analysis were used to measure the proteins and genes of the AKT/Phospho-ß-catenin (S552) pathway and autophagy. Apoptosis was measured by flow-cytometry.

**Results:**

We found that BAMLET significantly reduced cell viability and decreased the expression of CK1α. Additionally, BAMLET inhibited autophagy flux and enhanced the ability of CK1α inhibitor D4476 to impair autophagy flux, which was accompanied by an increase in the apoptosis percentage. We also observed that BAMLET empowered D4476 to down-regulate the AKT/Phospho-ß-catenin (S552) axis.

**Conclusion:**

BAMLET hampers autophagy flux and leads to apoptosis induction, possibly, by reducing the expression of CK1α and attenuation of the AKT/Phospho-ß-catenin (S552) axis.

## Introduction

Colorectal cancer (CRC) is the third most recognized cancer, killing 1 million people each year (1). CRCs usually develop as a result of stepwise, multiple mutations involving oncogenes and tumor suppressor genes (2). Oncogenic RAS activation is one of the prevalent mutations that occur in approximately 50% of CRCs (3). Activated RAS is associated with increased proliferation, angiogenesis, and motility, as well as with decreased apoptosis (4). RAS-driven cancer cells also up-regulate autophagy machinery under starvation or stress conditions (5, 6). Autophagy is the innate, regulated mechanism that isolates damaged organelles and misfolded proteins, and directs them to lysosomes for degradation to sustain homeostasis (7). Several studies have reported that autophagy is an essential mechanism for RAS-mutated cancer cells because it supports their metabolism and survivability (8, 9). However, aberrant up-regulation of autophagy might also result in cell death, called programmed cell death type II (10). Since it is currently impossible to directly target the RAS protein (11), identifying and targeting key molecular mechanisms that are involved in autophagy regulation in RAS-mutated cancer cells might be therapeutically useful. 

Recently, researchers have shown that casein kinase 1 alpha (CK1α) is the main regulator of autophagy flux in cancer cells harboring RAS mutations (12). CK1α is a serine/threonine-protein kinase that gets involved in different cellular processes, such as cell division, Wnt/β-catenin signaling pathway, membrane transport, nuclear localization, DNA repair, and gene transcription (13). CK1α also controls protein kinase B (AKT) activity in multiple myeloma (14). Activated AKT regulates cellular activity by phosphorylating a range of intracellular proteins (15). It has been reported that upon phosphorylation of β-catenin (a transcription factor that participates in the Wnt signaling pathway) by AKT at Ser 552, β-catenin translocates into the nucleus which increases colon cancer metastasis (16). Notably, evidence has demonstrated that β-catenin negatively regulates autophagy in different tumor cells (17). In our recent work, we demonstrated that CK1α inhibition suppresses autophagy flux possibly through down-regulating AKT/Phospho-ß-catenin (S552) pathway in human RAS-mutated CRC HCT116 cells (18). Therefore, exploring compounds with the ability to target CK1α could be effective in killing RAS-mutated tumor cells.

It has previously been observed that the HAMLET (Human α-lactalbumin Made Lethal to Tumors) compound targets a wide range of protein kinases including the CK1 family (19). HAMLET is a compound of oleic acids and a-lactalbumin that kills tumor cells (20). The bovine counterpart of HAMLET has been called BAMLET (Bovine α-lactalbumin Made Lethal to Tumors) which is also cytotoxic to cancer cells (21). In addition to *in vitro* studies, the anti-tumor effect of this lipid-protein complex has been confirmed in various *in vivo* investigations, such as nude rats with human brain tumor xenografts (22), human bladder cancer (23), colon cancer in the APCMin/+mice (24), and patients with skin papillomas (25). 

Therefore, this study aimed to investigate the effects of BAMLET on CK1α expression, AKT/Phospho-ß-catenin (S552) pathway, and autophagy flux in RAS-mutated human colorectal HCT 116 cells.

## Materials and Methods

***Chemical reagents and antibody***

Antibodies against human p62 (88588S), AKT (4691S), p-AKTS473 (9271S), p-β-cateninS552 (9566S), and CK1 (2655S) were purchased from Cell Signaling Technology (Beverly, MA, USA); 3-(4,5-dimethylthiazol-2-yl)-2,5-diphenyltetrazoliumbromide (MTT) (M2128), α-Lactalbumin from bovine milk (L6010), Oleic acid (O1383), Anti-rabbit IgG, anti-mouse IgG, and LC3B antibody (L7543) were purchased from Sigma-Aldrich (St. Louis, MO, USA); Glycerinaldehyde-3-phosphate-dehydrogenase (GAPDH) (sc-47724) and β-catenin (sc-133238) antibodies were purchased from the Santa Cruz Biotechnology (California, USA). CK1α inhibitor D4476 (ab120220) was purchased from Abcam (Cambridge, MA, USA). 

***Cell Culture***

Human colorectal (HCT116) carcinoma cells were obtained from the National Cell Bank of Iran Pasteur Institute. Cells were cultured at 37 ^°^C in a humidified atmosphere of 95% air and 5% CO_2_ in RPMI-1640 medium (Invitrogen, Carlsbad, CA, USA) containing 1% streptomycin (Gibco, USA) and 10% Fetal Bovine Serum (FBS)(Gibco™; Cat #: 16000044).

***Production of BAMLET***

BAMLET was produced according to the procedure used by Kamijima *et al*. Briefly, α-Lactalbumin was dissolved at 700 µM in phosphate-buffered saline (PBS). After the addition of oleic acid (120 molar equivalents) to the protein solution, the mixture was kept at 50 ^°^C for 10 min. The mixture was cooled to 25 ^°^C and centrifuged at 10,000× g for 10 min. Then the excess oleic acid was removed via aspiration and after dialysis for 18 hr, the complexes were stored at -20 ^°^C (26).

***Protein assessment***

Protein was assessed by BCA Protein Assay Kit (Novagen, San Diego CA, USA) according to the manufacturer’s recommendations.

***MTT assay***

MTT test was done according to the procedure of Ghavami *et al*. Briefly, HCT116 cells were seeded in 96-well plates at a density of 12000 cells/ml and incubated with varying concentrations of BAMLET (0-600 µg/ml) for 24 hr. Then, 20 μl of MTT (5 mg/ml) was added to each well. After 4 hr at 37 ^°^C, the medium was removed. To solubilize the generated formazan crystals, 200 μl of DMSO was added to each well. Finally, the absorbance of the solubilized formazan crystals was measured at 570 nm (27).

***Western blot analysis***

Immunoblotting was carried out to determine β-catenin, p-β-cateninS552, AKT, p-AKTS473, CK1α, p62, LC3βII, and GAPDH. The protein levels were normalized to GAPDH, as an endogenous control. Analysis was done according to the procedure of Alizadeh *et al*. (28). Total cell proteins were prepared by lysis in NP40 buffer containing 0.5% Nonidet P-40, 20 mM Tris-HCl (pH 7.5), a phosphatase inhibitor, 0.5 mM PMSF, protease inhibitor cocktails (Sigma, Cat#: P8340), and 100 μM β-glycerol 3-phosphate. After 8 min of centrifugation (10,000× g), the total cell protein lysates were quantified by the BCA protein assay kit (Novagen, San Diego CA, USA). Lysates were heated and separated by sodium dodecyl sulfate-polyacrylamide gel electrophoresis (SDS-PAGE). Afterward, proteins were transferred to the nitrocellulose membranes, the membranes were blocked and incubated with specified primary antibodies. After washing, blots were incubated with appropriate HRP-conjugated secondary antibody at RT for 2 hr. Then, blots were washed and incubated with enhanced chemiluminescence (ECL) reagents (ab133406). The signals were detected by ChemiDoc TM MP System (BIO-RAD, USA) and quantified, using the densitometry software Image Lab.

***Real-time PCR***

To extract the total RNA, cultured cells were exposed to the BIOZOL RNA extraction reagent (BSC51M1, Zhejiang, China). Then, Single-stranded cDNA was synthesized from the total RNA, using the Fermentase cDNA Synthesis Kit (USA). Consequently, the mRNA level of target genes was measured by a 7500 Real-time PCR system (Applied Biosystems, USA), using SYBR Green. The mRNA level of the CK1α, LC3B, p62, AKT-1, AKT-2, and β-catenin genes was normalized to GAPDH, as an endogenous control. The 2^-^^△△^^Ct^ formula was used to calculate the relative amount of mRNA in each reaction. Table 1 presents the primer pairs that were used for Quantitative Real-time PCR.

***Flow-cytometry***

The Nicoletti method was used to assess apoptosis (29). Accordingly, HCT116 cells were cultured in 6-well plates and exposed to BAMLET (100 µg/ml and 200 µg/ml), CK1α inhibitor D4476 (5 μM), or with the combination of the two for 24 hr. Cells were trypsinized and detached via centrifugation at 1500× g for 5 min at 4 ^°^C. Then, after washing with cold PBS, a hypotonic propidium Iodide (PI) buffer (0.1% Triton X-100, 1% sodium citrate, 0.5 mg/ml RNase A, 40 μg/ml PI) was used for staining of cells. Finally, after incubation at 37 ^°^C for 30 min, the cell nuclei were measured by flow cytometry. Apoptotic nuclei were located behind the G1 peak and contained hypo-diploid DNA.

***Statistical analysis***

Statistical analysis was performed using GraphPad Prism, version 6 (GraphPad Software). One-way ANOVA followed by Tukey’s multiple comparisons test was carried out to assess the comparisons. A *P*-value <0.05 was considered significant.

## Results

***BAMLET reduces the expression of CK1α and inhibits autophagy flux in HCT116 cells***

**Figure 1:**
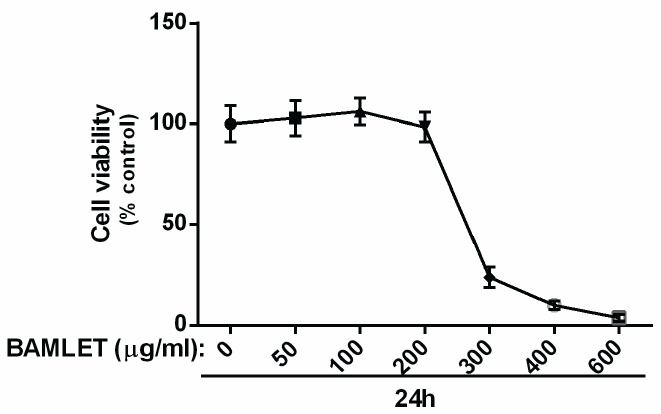
Effects of BAMLET (bovine α-lactalbumin made lethal to tumor cells) on cell viability in HCT116 cells

**Figure 2:**
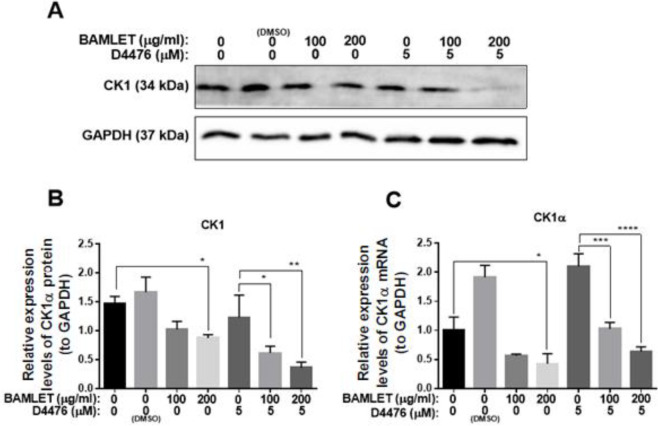
Effects of BAMLET (bovine α-lactalbumin made lethal to tumor cells) on CK1α expression in HCT116 cells

**Figure 3:**
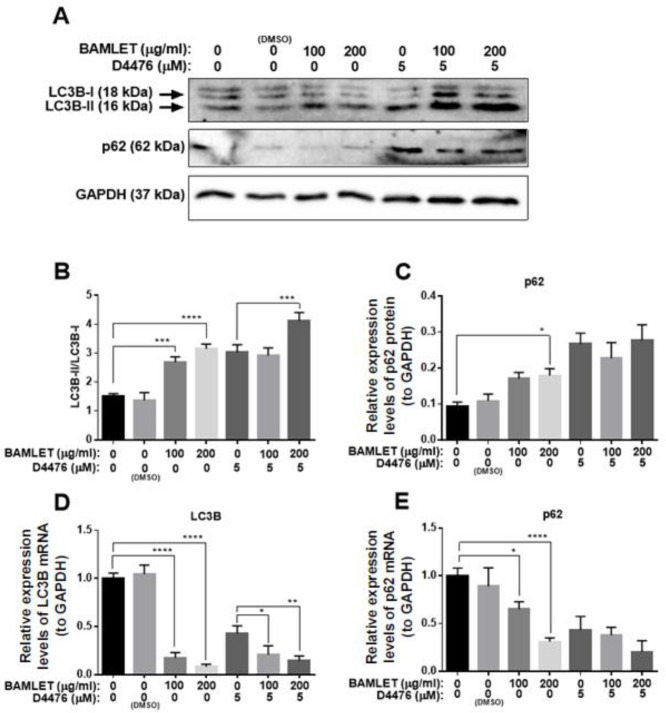
Effects of BAMLET (bovine α-lactalbumin made lethal to tumor cells) on autophagy flux in HCT116 cells

**Figure 4:**
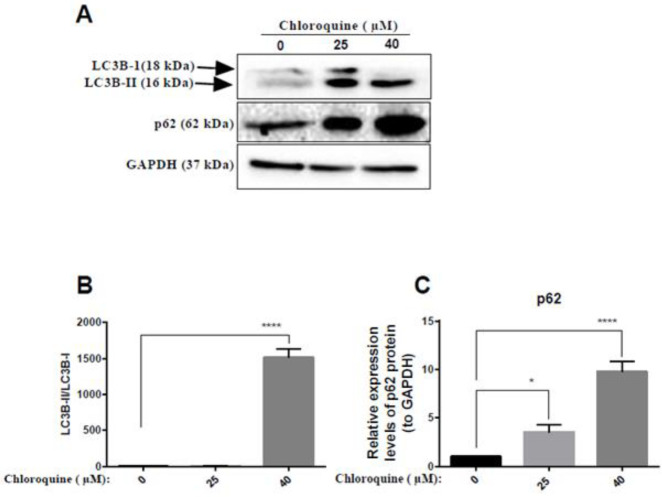
Effects of chloroquine on autophagy flux in HCT116 cells

In order to study the effect of BAMLET on CK1α expression and autophagy flux in the HCT 116 cell line, firstly, we performed an MTT assay to choose appropriate doses for the treatment. Our MTT data demonstrated that BAMLET we made could reduce the cell viability of HCT116 cells dose-dependently (IC_50_=250 µg/ml) (Figure 1). In a separate set of experiments, total RNA and protein were harvested for Real-time PCR and immunoblotting after treatment with BAMLET (100 µg/ml and 200 µg/ml), CK1 inhibitor D4476 (5 µM) or with the combination of the two for 24 hr. As shown in Figure 2A-2C, BAMLET decreased the CK1α expression at both mRNA and protein levels. The expression of CK1α was also reduced in the combination groups compared with cells that were solely treated with D4476 (Figure 2A-2C). Then, we explored the effect of BAMLET on autophagy flux in HCT116 cells, using antibodies against LC3 and p62 proteins as well-known markers of autophagy. Upon activation of autophagy, LC3B is hydrolyzed to create the cytosolic LC3B-I. After lipidation, LC3B-I turns to LC3B-II, which is embedded in the autophagosome membrane. Finally, the LC3B-II binding with p62 promotes the fusion of the autophagosome with lysosomes and encourages the clearance of ubiquitinated proteins (7). We found that BAMLET significantly induced dose-dependent LC3β lipidation, while reducing the level of its mRNA (Figures 3A, 3B, and 3D). Moreover, BAMLET increased the level of p62 protein (Figures 3A and 3C), while significantly decreasing the level of its mRNA (Figure 3E). We also observed that BAMLET potentiated the suppression of autophagy flux induced by D4476 which is indicated by increase in the levels of LC3β lipidation and p62 protein and decrease in their mRNA levels (Figure 3A-3E). Moreover, the autophagy inhibitor chloroquine was used to confirm autophagy flux inhibition. Chloroquine increases lysosomal pH which leads to inhibition of autophagy flux and lysosomal protein degradation. Our western blot data indicated that LC3β lipidation and p62 protein levels were increased in chloroquine-treated cells, corroborating the inhibition of autophagy flux by chloroquine (Figures 4A, 4B, and 4C). Altogether, these results show that BAMLET down-regulates CK1α and inhibits autophagy ﬂux in HCT116 cells.

***BAMLET induces apoptotic cell death in HCT116 cells***

**Figure 5.**
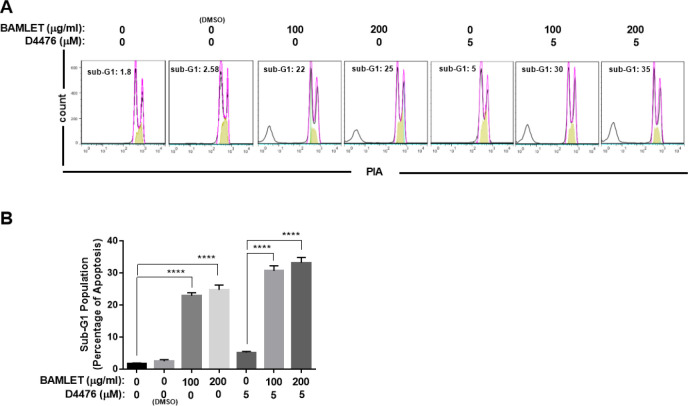
BAMLET (bovine α-lactalbumin made lethal to tumor cells) leads to apoptosis in HCT116 colorectal cancer cell line

To further examine whether BAMLET-mediated autophagy flux inhibition eventually affected HCT116 cell survival, apoptotic cell death was analyzed. As can be seen from Figures 4A and 4B, BAMLET significantly increased the percentage of apoptotic cells in a dose-dependent manner. Our results also demonstrated that the combination of BAMLET and D4476 significantly enhanced the percentage of sub-G1 abundance, compared with cells treated with D4476 alone (Figures 5A and 5B).

***BAMLET down-regulates the AKT/p-ßcateninS552 axis in HCT116 cells***

**Figure 6:**
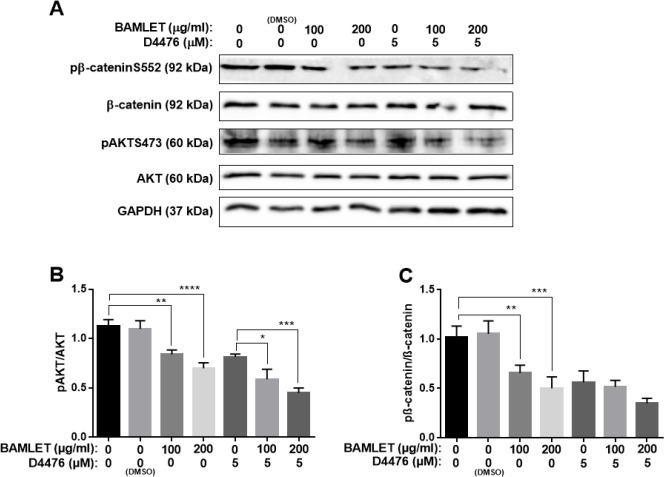
Effects of BAMLET (bovine α-lactalbumin made lethal to tumor cells) on AKT/ pβ-cateninS552 axis in HCT116 cells

**Figure 7:**
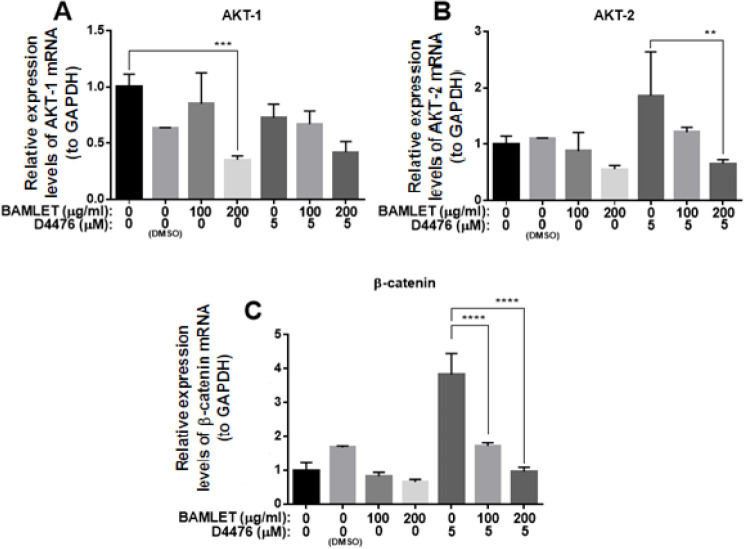
Effects of BAMLET (bovine α-lactalbumin made lethal to tumor cells) on AKT-1, AKT-2, and β-catenin gene expression in HCT116 cells

We have recently shown that CK1α controls autophagy machinery possibly through the AKT/p-ßcateninS552 signaling in RAS-mutated human Colorectal HCT 116 cells (18). Therefore, to get a deeper insight into the underlying mechanism of autophagy flux inhibition which was triggered by BAMLET, the expression of the AKT/p-ßcateninS552 axis was measured using the Western Blot analysis and Real-time PCR. Figure 6A-6C illustrates that BAMLET significantly declined the p-AKT level and its substrate, ß-catenin. We also found that BAMLET empowered D4476 to reduce the p-AKT and ß-catenin (Figure 6A-6C). Consistent with western blot results, our real-time PCR data also indicated that BAMLET and co-treatment with BAMLET and D4476 reduced the mRNA levels of AKT1, AKT2, and ß-catenin (Figure 7A-7C). All in all, these findings demonstrated that BAMLET attenuates the AKT/p-ßcateninS552 axis in HCT116 cells.

**Table 1:** Primer sequence used for Quantitative Real-time PCR

Gene	Forward primer	Reverse primer
GAPDH	5′-CGACCACTTTGTCAAGCTCA-3′	5′-AGGGGTCTACATGGCAACTG-3′
AKT-1	5′ -TTGTTATTGTGTATTATGTTGTTCA- 3′	5′ -AAGTGCTACCGTGGAGAG-3′
AKT-2	5′ -CCTTAAACAACTTCTCCGTAGCA-3′	5′ -GCAGGCAGCGTATGACAAA-3′
P62	5′ -AATCAGCTTCTGGTCCATCG-3′	5′-TTCTTTTCCCTCCGTGCTC-3′
LC3B	5′-AACGGGCTGTGTGAGAAAAC-3′	5′-AGTGAGGACTTTGGGTGTGG-3′
CK1α	5′-AATGGGTATTGGGCGTCACTGTAA-3′	5′-CCTGAGAAAGATGGGTCCTGAGAA-3′
β-catenin	5′-AAAATGGCAGTGCGTTTAG-3′	5′-TTTGAAGGCAGTCTGTCGTA-3′

The cells were treated with different concentrations (0-600 µg/ml) of BAMLET for 24 hr. Cell viability was evaluated by MTT assay and reported as percentage of treated cells

HCT116 cells were treated with BAMLET (100 µg/ml and 200 µg/ml), CK1α inhibitor D4476 (5 µM), or with a combination of the two for 24 hr. (A) CK1 protein levels were assessed by immunoblotting. (B) Semiquantitative density analysis of CK1. CK1 protein levels were normalized to GAPDH protein levels. (C) Relative expression levels of CK1α mRNA. CK1α mRNA levels were normalized to GAPDH mRNA levels. One-way ANOVA followed by Tukey’s multiple comparisons test was carried out to assess the comparisons in B and C; *P<0.05; ***P<0.001; ****P<0.0001

HCT116 cells were treated with BAMLET (100 µg/ml and 200 µg/ml), CK1α inhibitor D4476 (5 µM), or with a combination of the two for 24 hr. (A) LC3B and p62 protein levels were assessed by immunoblotting. (B) Semiquantitative density analysis of LCB-ΙΙ to LC3B-I. (C) Semiquantitative density analysis of p62. p62 protein levels were normalized to GAPDH protein levels. (D) Relative expression levels of LC3B mRNA (E) Relative expression levels of p62 mRNA. One-way ANOVA followed by Tukey’s multiple comparisons test was carried out to assess the comparisons in B, C, D, and E; *P<0.05; **P<0.01; ***P<0.001; ****P<0.0001

HCT116 cells were treated with chloroquine (25 µM and 40 µM) for 24 hr. (A) LC3B and p62 protein levels were assessed by immunoblotting. (B) Semiquantitative density analysis of LCB-ΙΙ to LC3B-I. (C) Semiquantitative density analysis of p62. p62 protein levels were normalized to GAPDH protein levels. One-way ANOVA followed by Tukey’s multiple comparisons test was carried out to assess the comparisons in B and C; *P<0.05; ****P<0.0001

Quantification of sub-G1 phase with PI staining and FACS analysis in HCT116 cell line, following 24 hr treatment with BAMLET (100 µg/ml and 200 µg/ml), CK1α inhibitor D4476 (5 µM) or with the combination of the two. (A) Representative FACS histogram plot. PI, propidium iodide. (B) Percent sub-G1 abundance induced by D4476 treatment after 24 hr. One-way ANOVA followed by Tukey’s multiple comparisons test was carried out to assess the comparisons in B; ****P<0.0001

HCT116 cells were exposed to BAMLET (100 µg/ml and 200 µg/ml), CK1α inhibitor D4476 (5 µM), or the combination of the two for 24 hr. (A) pAKT, AKT, pβ-catenin, and β-catenin protein levels were assessed by immunoblotting. (B, C) Semiquantitative density analysis of pAKT/AKT and pβ-catenin/β-catenin. One-way ANOVA followed by Tukey’s multiple comparisons test was carried out to assess the comparisons in B and C; *P<0.05; **P<0.01; ***P<0.001; ****P<0.0001

HCT116 cells were exposed to BAMLET (100 µg/ml and 200 µg/ml), CK1α inhibitor D4476 (5 µM), or the combination of the two for 24 hr. (A) Relative expression levels of AKT-1 mRNA. (B) Relative expression levels of AKT-2 mRNA. (C) Relative expression levels of β-catenin mRNA. One-way ANOVA followed by Tukey’s multiple comparisons test was carried out to assess the comparisons in A, B, and C; **P<0.01; ***P<0.001; ****P<0.0001

## Discussion

Activating mutations in RAS oncogene play a key role in CRC cell growth and promote resistance to anti-EGFR (epidermal growth factor receptor) therapy (30). They are also associated with poor prognosis in colon cancer cases. To date it has been impossible to directly target RAS oncoprotein, in this regard, researchers suggested that agents with the ability to target downstream signaling pathways of RAS could be therapeutically useful (11). 

Here in the present study, we showed that a lipid-protein complex named BAMLET induced apoptosis in RAS-mutated human colorectal HCT 116 cells through inhibition of autophagy mechanism and down-regulation of protein kinase CK1α. Autophagy is the innate, regulated mechanism that is activated in conditions such as stress, starvation, chemotherapy, and organelle damage (31). Cancer cells usually up-regulate autophagy flux due to high metabolic stress (32). Based on tumor type, stage, and cellular context, autophagy may have anti- or pro-tumorigenic effects (33). The effects of diverse oncogenes and tumor suppressors on autophagy have been explained in a great number of studies (34). For instance, it has been reported that colon cancer cells with RAS mutation up-regulate autophagy through activation of the MEK/ERK pathway (35). In this case, autophagy aids CRC cells’ survival during starvation. Additionally, Brent *et al*. demonstrated that autophagy prevents RAS-driven CRC cell apoptosis and contributes to proliferation (36). The positive role of autophagy in tumorigenesis was also described in other types of RAS-mutated tumor cells such as non-small cell lung cancer (37), melanoma (38), and pancreatic tumor cells (39). Although autophagy supports tumor cells’ survival and progression, paradoxically, uncontrolled autophagy activation leads to cell death, called programmed cell death type II (40). This type of cell death morphologically is characterized by the accumulation of a large number of autophagosomes in the cytoplasm (41). Aberrant autophagy activation and lysosomal dysfunction are two major reasons for the accumulation of autophagosomes and thereby autophagy flux inhibition (10). LC3B-II and p62 are well-known autophagy markers whose levels increased during autophagy flux suppression (42). Consistent with this, our results demonstrated that protein levels of LC3B-II and p62 significantly increased in response to BAMLET treatment. However, our real-time PCR data demonstrated that mRNA levels of LC3B and p62 significantly decreased during BAMLET treatment. A possible explanation for this might be that accumulated LC3B-II and p62 proteins down-regulate their mRNA through a negative feedback loop mechanism. Previous studies also reported that autophagy is involved in cell death induced by HAMLET (the human counterpart of BAMLET) (43). Researchers reported that accumulation of p62 protein in response to HAMLET could promote apoptosis through caspase-8 activation (43). Besides, it has been found that an excessive increase in autophagosomes leads to cell death possibly by inhibiting the degradation of misfolded proteins or by inducing a cell death signal (12). Recently, Cheong *et al*. indicated that RAS-mutated cancer cells prevent excessive autophagy activation through up-regulation of protein kinase CK1α (12). They showed that inhibition of CK1α leads to aberrant autophagy activation and thereby accumulation of ineffective autophagic vesicles which in turn induce cell death. The modulatory role of CK1α on autophagy was also described in other tumor cell types including acute myeloid leukemia (44), lung tumors (45), osteosarcoma, and neuroglioma (46). In the current study, we showed that BAMLET reduced CK1α expression both at mRNA and protein levels in RAS-mutated human colorectal HCT 116 cells. This data is in line with Ho’s (2016) findings which demonstrated that HAMLET targets a wide range of protein kinases including the CK1 family (19). Furthermore, our data demonstrated that BAMLET empowers CK1 inhibitor D4476 to suppress autophagy flux and promote apoptosis. Therefore, it seems that BAMLET inhibits autophagy flux and thereby induces apoptosis through down-regulation of CK1α. 

Next, we investigated the mechanism by which down-regulation of CK1α impairs autophagy flux in response to BAMLET treatment. Our recent *in vitro* data suggest that CK1α inhibits autophagy flux possibly through AKT/Phospho-ß-catenin (S552) pathway in colon cancer cells with RAS mutation (18). ß-catenin is the main transcription factor in Wnt/ß-catenin signaling that controls the expression of an array of genes contributing to cell proliferation, metastasis, drug resistance, and cell survival (47). Additionally, recent studies noted that autophagy machinery is under negative control of the Wnt/β-catenin signaling pathway (48). For example, a study reported that βcatenin silencing in multiple myeloma cells leads to an increase in the number of autophagosomes and up-regulation of the LC3B and Beclin1 genes (49). Beta-catenin stabilization is the signature of the Wnt/β-catenin pathway (50). In the nucleus, β-catenin via binding with TCF/LEF (T cell-specific transcription factor/lymphoid enhancer-binding factor 1) regulates the target gene expression (51). In addition to Wnt ligands, the AKT pathway also leads to stabilization of β-catenin (52). Several studies have indicated that β-catenin phosphorylation at Ser552 by the AKT pathway leads to its accumulation in the nucleus and increases its transcriptional activity (53). The mammalian target of rapamycin (mTOR) activates AKT via phosphorylating Ser473 (54). Additionally, it has been observed that DEPTOR (DEP-domain containing mTOR-interacting protein) inhibition by CK1a leads to activation of mTOR (55). Accordingly, our group has reported that CK1a inhibition attenuates the AKT/phospho-β-catenin (S552) signaling in CRC cells harboring RAS mutation which was associated with autophagy flux inhibition and induction of apoptosis (18). Similarly, in this work, we observed that BAMLET down-regulates the AKT/phospho-β-catenin (S552) axis and enhances the ability of CK1a inhibitor to decrease the expression of the AKT/phospho-β-catenin (S552) signaling in CRC cells harboring RAS mutation. Altogether, these results suggest that BAMLET weakens the AKT/phospho-β-catenin (S552) signaling pathway via down-regulation of CK1a and eventually leads to autophagy flux inhibition and cell death.

## Conclusion

In the current study, we investigated the effects of BAMLET on CK1α expression, autophagy flux, and AKT/Phospho-ß-catenin (S552) pathway in RAS-mutated human colorectal HCT 116 cells. In summary, our data indicated that BAMLET impairs autophagy flux and leads to apoptosis induction in RAS-mutated human colorectal HCT 116 cells possibly via down-regulating of CK1α and attenuation of AKT/p-β-cateninS552 signaling. These findings suggest that BAMLET could be used for the targeting of CRC that is caused by RAS activation.

## Authors’ Contributions

P M designed the research, reviewed the paper, and contributed to supervision, resources, and funding acquisition of the study. H B performed the experiments and analyzed data and wrote the paper. All authors have read and approved the manuscript.

## Conflicts of Interest

The authors report no conflicts of interest. 
